# Comparing outcomes following direct admission and early transfer to specialized trauma centers in open tibial fracture treatment: a systematic review and meta-analysis

**DOI:** 10.1007/s00068-023-02366-x

**Published:** 2023-09-30

**Authors:** Pien Gabriele Francien Joosten, Marieke Paulina Borgdorff, Matthijs Botman, Mark-Bram Bouman, Daphne van Embden, Georgios Fredericus Giannakópoulos

**Affiliations:** 1 Trauma Unit, Department of Surgery, Amsterdam University Medical Center, Meibergdreef 9, 1105AZ Amsterdam, The Netherlands; 2 Department of Plastic, Reconstructive, and Hand Surgery, Amsterdam University Medical Center, Meibergdreef 9, J1A-207, 1105AZ Amsterdam, The Netherlands; 3 Amsterdam Movement Sciences, Musculoskeletal Health, Amsterdam, The Netherlands

**Keywords:** Open fracture, Lower extremity, Orthoplastic, Tertiary care centers, Complications

## Abstract

**Introduction:**

Guidelines on the management of open tibia fractures recommend timely treatment in a limb reconstruction center which offer joint orthopedic-trauma and plastic surgery services. However, patient’s transfer between centers remains inevitable. This review aims to evaluate the clinical outcomes and hospital factors for patients directly admitted and transferred patients to a limb-reconstruction center.

**Methods:**

A research protocol adhering to PRISMA standards was established. The search included databases like MEDLINE, EMBASE, and the Cochrane library up until March 2023. Nine articles met the inclusion criteria, focusing on open tibia fractures. Exclusion criteria were experimental studies, animal studies, and case reports. Outcomes of interest were operation and infection rates, nonunion, limb salvage, and the Enneking limb score.

**Results:**

The analysis involved data from 520 patients across nine studies published between 1990 and 2023, with the majority (83.8%) having Gustilo Anderson type III open tibia fractures. Directly admitted patients showed lower overall infection rates (RR 0.30; 95% CI 0.10–0.90; *P* = 0.03) and fewer deep infections (RR 0.39; 95% CI 0.22–0.68; *P* = 0.001) compared to transferred patients. Transferred patients experienced an average five-day delay in soft tissue closure and extended hospital stays by eight days. Patients transferred without initial surgical management underwent fewer total surgical procedures. The direct admission group displayed more favorable functional outcomes.

**Conclusion:**

Low- to moderate-quality evidence indicates worse clinical outcomes for transferred patients compared to directly admitted patients. Early treatment in specialized limb reconstruction units is essential for improved results in the management of open tibia fractures.

**Level of evidence:**

Therapeutic level IIa.

## Introduction

One of the most common types of open fractures is the open tibial fracture, accounting for more than 11% of all open fractures [1]. Open fractures are internationally recognized by the Gustilo Anderson (GA) classification ranging from grade 1 (equal to a puncture wound) to grade 3C (with concomitant arterial injury). Open tibial fractures tend to be more severe, with the highest prevalence of Gustilo Anderson (GA) type III due to a relative lack of soft tissue coverage, imposing a high risk of developing complications and secondary amputation [1–3]. Despite the relatively low overall incidence in high-income countries, open tibia fractures are costly for patients, society, and the healthcare system [4]. If complications such as infection arise, delayed recovery may result in persistent disability [5]. Therefore, timely treatment by a specialized team is important, as appropriate fracture care and early soft tissue closure decrease the risk of infection and nonunion [6, 7].

Medical infrastructure is an important topic in severe extremity trauma, as since 2009, The British Association of Plastic, Reconstructive, and Aesthetic Surgeons (BAPRAS) and British Orthopaedic Association (BOA) guideline has included the consensus statement that patients with open lower extremity fractures should be treated in centers with a specialized multidisciplinary team [8–10]. These centers facilitate an orthoplastic service with scheduled daytime operation sessions, moving away from delays in soft tissue coverage and secondary referrals. The implementation of these guidelines has been shown to lead to decreased treatment duration and higher success rates, associated with reduced complications and consequent healthcare costs [11–14]. However, divergence between clinical treatment guidelines and clinical practice suggests that adherence to the guidelines is not always feasible [15–17]. The organization of a serviceable orthoplastic center is challenging. It requires combined services, sufficient operating lists, and outpatient clinics with specialized plastic surgery consultants availability to meet the timely treatment standards. Centralization of care for severe open tibia fractures is a topic of discussion, as specialization and high surgeon volume are associated with improved patient outcomes [18]. Additional changes to health policy require more high-level evidence on the specific care processes and patient outcomes following referral versus primary centralized treatment.

In order to establish current insights into the impact of transfer on open tibia fracture management and outcomes, we performed a systematic review of the literature. The aim was to provide an overview of hospital factors, success rates, and complications after direct admission (DAP), early (‘hot’) transfer (HTP), and general transfer to specialized orthoplastic centers (GTP).

## Materials and methods

### Literature search strategy

A research protocol was established in accordance with the PRISMA standards (Preferred Reporting Items for Systematic Reviews and Meta-Analysis) [19]. The search terms were designed to match all articles containing information on outcomes in the treatment of open tibial fractures in adults (16 years and older). A pre-selection in language was set to Dutch, German, and English. Experimental studies, studies on animals, and case reports were excluded.

### Study selection

**Fig. 1:**
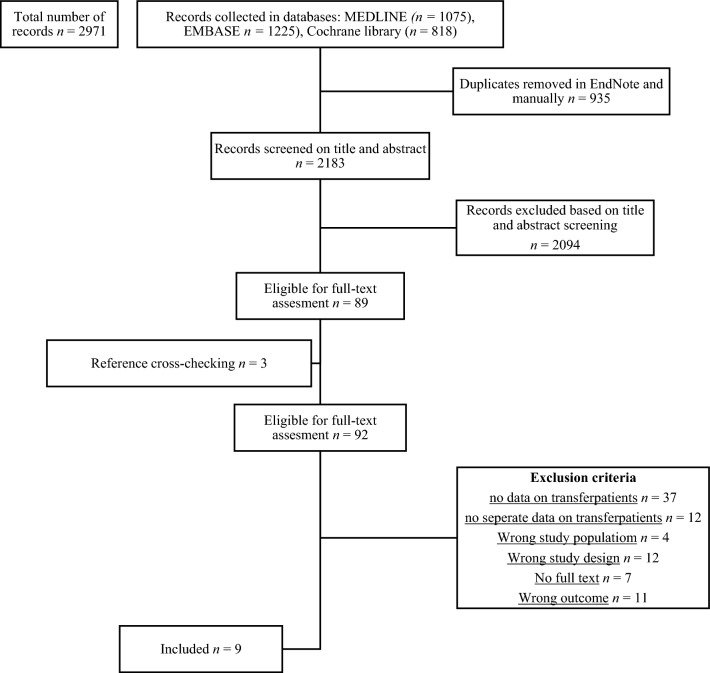
Prisma (preferred Reporting Items for Systematic Meta-Analyses). Study selection and inclusion flow diagram

In collaboration with a medical information specialist, we searched for records in MEDLINE, EMBASE, and the Cochrane library. The search was initiated in March 2020 and re-run in March 2023. Records were included from the first of January 1990 to the 20th of March 2023. A total of 4.057 records were exported to EndNote deduplication and article and abstract screening was done with Rayyan [20] (Fig. 1). Two authors screened all records independently. By cross-checking the references, three more articles were added.

Studies on the outcomes of open tibial fractures in relation to the center of treatment and inter-hospital transport were included if they contained any information on the clinical and/or functional outcomes. All types of open tibial fractures were included, with the exception of pathological fractures or underlying disease. Any final discrepancies in definitive inclusion were solved by a third senior author. Studies were excluded if they had no (separate) data on transferred patients, the cohort consisted of minors under the age of 16, included other fracture locations with inseparable data, they did not provide information on outcomes or circumstances or if no full text was available.

### Data extraction

One reviewer independently extracted the data from the included studies. The extracted data were checked by a second investigator. The following data were obtained:

1. Author, publication date, study design, and number of included patients.
2. Patient and treatment characteristics: gender, age, number of fractures, GA classification, type and location of the injury, mechanism of injury, length of hospital stay, time to transfer, time to soft tissue closure, number of procedures, unscheduled additional surgeries.
3. Clinical and functional outcomes: overall complications, superficial infections, deep infections, osteomyelitis, nonunion, malunion, secondary amputation, and functional scores.

The results were reported according to the three main groups found in the records, i.e., directly admitted patients to a specialized center (DAP), directly transferred patients who received surgical management solely in a limb-reconstruction center (in the literature used for this study referred to as hot-transfer patients, HTP) and transferred patients in general (GTPs) who received initial management elsewhere or whose status is unknown. If studies included mixed cohorts and individual patient data were not reported, the authors were first contacted by email to request the above-mentioned data. If the additional information was not obtainable, and we were not able to calculate the data from available data points, the article was excluded.

### Risk of bias

The risk of bias in the included studies was evaluated with the “Newcastle–Ottawa Quality Assessment Form for Cohort Studies” [21]. In this tool, eight questions are divided into the sections “selection”, “comparability” and “outcome”. Each question can be rewarded with a star; in the section “comparability”, a maximum of two stars can be rewarded. The total number of stars in each section correlates to a final assessment of quality as; poor, fair, or good. An article can earn a maximum of nine stars.

### Statistical analysis

Demographics were summarized using descriptive statistics. A meta-analysis was deemed possible if three or more articles were reported on the outcome of interest. Data on the outcomes were pooled using the random-effects model due to the differences in transfer times, treatment protocols, and potential baseline characteristics. The effect size of dichotomous data was reported using the relative risk ratio (RR) and 95% confidence intervals. Forest plots were used to report the effect summary of individual studies. If data were not available or incomplete for analysis, data were reported in the narrative. A *P *value of > 0.05 was deemed statistically significant. Data were analyzed using the Cochrane RevMan version 5.4 software and IBM SPSS statistics 28.

## Results

### Study selection

The search strategy is presented in a PRISMA diagram (Fig. 1). Nine studies met the inclusion criteria of this systematic review, published between 2005 and 2018. An overview of the study characteristics is summarized in Table 1.

**Table 1:** Summary of study characteristics

Study	Study design	No of patients (fractures)	DAP group (No of patients)	HTP group (No of patients)	GTP group (No of patients)	Mean age (years)	Gustilo-Anderson classification
Hendrickson (2018)	Retrospective cohort	112 (115)	72	NS	44	50	IIIB (100%)
Crowe (2017)	Retrospective cohort	34 (34)	17	NS	17	45	IIIB (100%)
Wordsworth (2016)	Prospective cohort	65 (66)	32	NS	23	42	IIIB (100%)
Trickett (2015)	Prospective cohort	35	8	7	27	NS	II–IIIC (54% IIIB or IIIC)
Chummun (2012)	Retrospective cohort	45	NA	17	45	42	IIIB (100%)
Townley (2010)	Prospective cohort	55 (55)	22	NS	33	37	I–IIIC (64% IIIB or IIIC)
Singh (2009)	Prospective cohort	36 (36)	13	NS	23	43	IIIB (100%)
Naique (2006)	Prospective cohort	72 (73)	25	NS	47	42	IIIB (100%)
Alisson (2005)	Retrospective cohort	66 (66)	33	NS	33	66	I–IIIC (44% IIIB or IIIC)

*DAP* direct admission patients, *HTP* hot transfer patients, *GTP* general transfer patients, *NA* not applicable, *NS* not specified

In total, 520 patients were included with 526 fractures. The average age of the included patients was 46.9 years (17–98 years), with the minority being female (37.4%). The majority were GA type III open fractures (*N* = 441, 83%), and 88% (*N* = 387) were reported as type IIIB. Seven articles reported the GA grade for separate admission groups (Fig. 1). Pooled analysis suggests that the risk of direct admission compared to transfer for GA type III open tibial fractures might not differ (RR, 0.98; 95% CI 0.91–1.06; *p* = 0.62; participants, 435; *I*^*2*^ = 85%). Road traffic accidents were the overall predominant cause of injury (58.2%), followed by falls in 28.8%. Patient details are listed in Table 2.

**Table 2:** Demographic overview per treatment group

Group	DAP	HTP	GTP
No of fractures	222	17	292
Mean age	46.3	43.6	42.7
% male	71%	47%	67%
Gustilo-Anderson classification *N *(% per group)			
Gustilo I	5 (5.6)	–	3 (2)
Gustilo II	14 (15.7)	–	2 (1.4)
Gustilo IIIA	6 (6.8)	–	7 (4.7)
Gustilo IIIB	60 (67.4)	17 (100)	133 (89.9)
Gustilo IIIC	4 (4.5)	–	3 (2)
Mechanism of injury *N* (% per group)			
Road traffic	11 (64.8)	9 (52.9)	19 (42.3)
Fall	4 (23.5)	7 (41.2)	23 (51.1)
Crush/ballistics	2 (11.8)	1 (5.9)	2 (4.4)
Sports	NA	NA	NA
Other	NA	NA	1 (2.2)

*DAP* direct admission patients, *HTP* hot transfer patients, *GTP* general transfer patients, *NA* not applicable

### Circumstances in inter-hospital transport

#### Time to transfer

Four studies reported the mean transfer time. The overall mean time to transfer was 6.85 days and ranged from 0 to 45 days. All four studies did not report the timing and location of the first debridement, as shown in Table 3.

**Table 3:** Circumstances in inter-hospital transport

Hospital data	DAP	HTP	GTP
Mean transfer time in days	NA	NA	4.6D
Length of hospital stay in days	21.8D	NA	30.1D
Time to soft tissue coverage	7.2D	NA	12.6D
Amount of procedures^a^	2.3	2.3	3.5
Revisions^a^	1.4	1.7	2.5

*DAP* direct admission patients, *HTP* hot transfer patients, *GTP* general transfer patients, *NA* not applicable

^a^Average per patient

#### Timing of soft tissue coverage

A total of three studies reported the average time to soft tissue coverage. Townley et al*.* reported 10.8 days (range 2–49) for TPs versus 3.6 days (range 0–15 days) for DAPs (*P* < 0.001) [22]. Two other studies [23, 24] showed a trend towards quicker coverage for DAP; however, they did not reach statistical significance. Another study showed 77% soft tissue coverage within five days in DAPs compared to 43% in TPs [24]. Chummun et al*.* showed an overall longer time to definitive surgery when comparing hot transfers to general transfers; 3.9 days versus 7.7 days (*P* = 0.03) [25]. See Table 3.

#### Length of hospital stay (LOHS)

Three studies reported on the total LOHS, meaning the combined duration of hospital admissions. GTPs were admitted to the hospital for a longer total period than DAPs (Table 5). The mean of 29.3 days for GTPs was compared to 21.8 days for DAPs [22, 23, 26], as shown in Table 3.

#### Total number of procedures and acute revisions

Seven studies reported on revisions, acute re-interventions, or total surgical procedures [6, 12, 22–26]. Trickett et al*.* reported significantly more procedures in the GTP group versus the DAP group within 30 days (*P* = 0.02) and 12 months (*P* = 0.05). They show an average of two procedures for DAPs versus 4.2 procedures for TPs within 12 months [12]. The GTP group underwent overall more revisions and procedures, as shown in Table 3.

#### Hot transfers

Trickett et al*.* showed that HTPs needed significantly fewer surgical interventions within 30 days (*P* = 0.0042) and 12 months (*P* = 0.0098), compared to GTPs who underwent surgical intervention [12]. Chummun et al*.* also demonstrated that HTP patients underwent significantly fewer surgeries than GTP patients (− 22%; 2.5 vs. 3.2 per patient; *P* = 0.003) [25].

### Outcomes in inter-hospital transport

#### Limb salvage

**Fig. 2:**
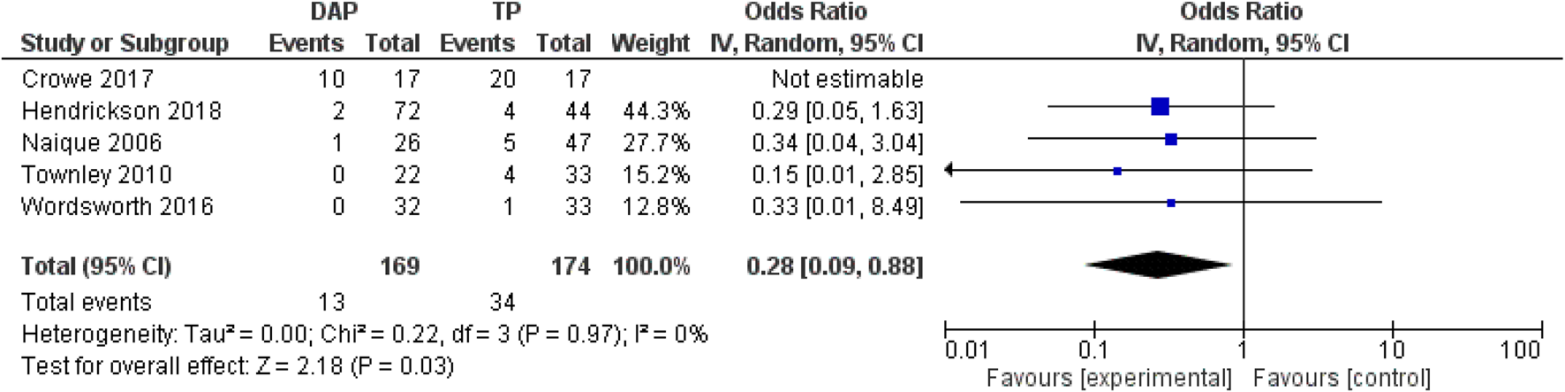
Random effect meta-analysis of the proportion of the overall infection rates in DAP and TP after open tibia fractures. Forest plots comparing DAP and GTP groups. Horizontal lines represent 95% CIs. *DAP* direct admission patients, *GTP* general transfer patients

Overall limb salvage after subtracting primary amputation was over 94% in the combined data of GTPs and DAPs (Table 5). None of the studies stated statistical significance between GTPs and DAPs in primary and secondary amputation. A pooled analysis of three papers suggests that the secondary amputation risk in DAP patients may be lower when compared to the GTP group; however, no statistically significant difference was found (RR, 0.64; 95% CI 0.14–2.95; *P* = 0.57; participants 172). See Fig. 2.

#### Infections

**Fig. 3:**
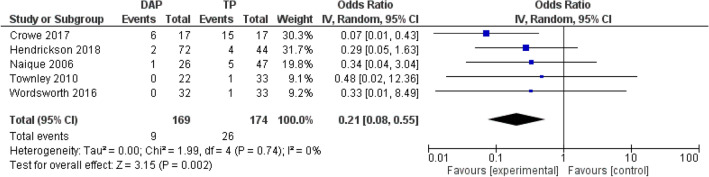
Random effect meta-analysis of the proportion of the deep infection rates in DAP and TP after open tibia fractures. Forest plots comparing DAP and GTP groups. Horizontal lines represent 95% CI. *DAP* direct admission patients, *GTP* general transfer patients

**Fig. 4:**
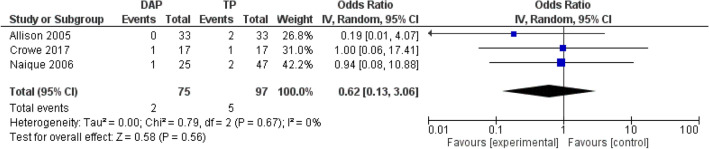
Random effect meta-analysis of the proportion of the secondary amputation rates in DAP and TP after open tibia fractures. Forest plots comparing DAP and GTP groups. Horizontal lines represent 95% CI. *DAP* direct admission patients, *GTP * general transfer patients

The study by Crowe et al*.* reported statistically significant reduced infection rates in the DAP group; three DAPs developed osteomyelitis compared to nine GTPs (*P* = 0.03) [23]. All other studies also showed that deep infections occurred more often in GTPs, though these rates did not account for statistically relevant results [13, 22, 26, 27]. Naique et al*.* stated that the highest rate of infection (16%) occurred in the GTP group debrided after six hours, compared to DAPs [6]. Pooled analysis from five studies suggests a lower risk of overall infections in the DAP group (RR, 0.30; 95% CI 0.10–0.90; *P* = 0.03; participants 343). Furthermore, pooled analysis of deep infection rates indicates a lower risk of deep infections compared to the GTP group (RR, 0.39; 95% CI 0.22–0.69; *P* = 0.001; participants 310). See Figs. 3 and 4.

#### Malunion and nonunion

One study reported separate malunion and nonunion rates for the GTP and DAP groups. The malunion rate was higher in the DAP group; three cases versus one case in GTP. The nonunion rate was higher in the GTP group: one case in DAP versus six in GTP [23], without statistical relevance (Table 4).

**Table 4:** Overview of outcomes in the different treatment groups

	Total *N* (%)	DAP *N* (%)	HTP *N* (%)	GTP *N* (%)
Clinical outcomes				
Overall infection rate	87 (19.4)	15 (10.8)	NA	52 (29.8)
Superficial infection	42 (15.5)	4 (10.3)	NA	8 (16)
Deep infection or osteomyelitis	37 (11.1)	9 (6.6)	NA	26 (14.9)
Mal-union	3 (8.5)	3 (17.6)	NA	1 (5.9)
Non and delayed union	35 (35)	9 (52.9)	NA	15 (88)
Primary amputation	8 (3.1)	5 (8.6)	NA	NA
Secondary amputations	10 (3.5)	2 (4.8)	NA	5 (5.2)
Enneking score %	77.2	75.0	81.5	76.9

*DAP* direct admission patients, *HTP* hot transfer patients, *GTP* general transfer patients, *NA* not applicable

#### The Enneking limb score

Two studies reported functional outcomes scores. The Enneking limb score was calculated in two articles, showing the best outcomes in the HTP group (81.5 versus 75 in the DAP group), as shown in Table 5 [6, 25] (Table 4).

**Table 5:**
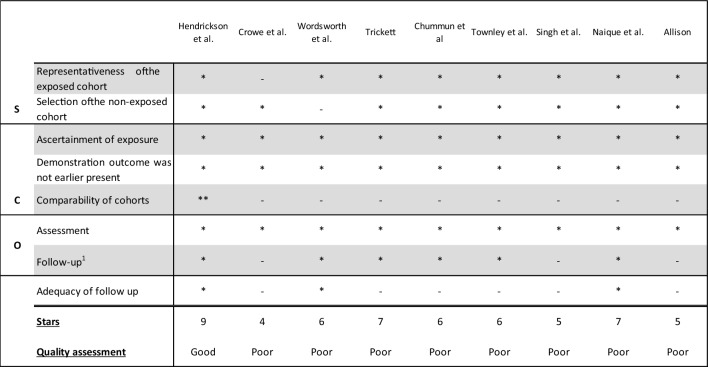
Methodological quality assessment using the Newcastle–Ottawa Quality Assessment Form for Cohort Studies

Due to heterogeneity of the literature no standard was set for follow up. The follow-up period was set to each article individually by regarding the aim of the study. Studies observing outcomes in open tibial fractures had a minimum follow-up set to 7.5 months as is the average time to union (42)

### Methodological appraisal

The overall quality evaluation of the articles was assessed as poor, although the assessment varied between five and nine stars (Table 2). The studies mainly qualified as poor due to a low score in comparability. Only one article scored stars in this section by adjusting their outcomes for general confounders such as age or specific confounders like the injury severity score. Most articles stated that there was no statistically significant difference in age and sex between the two cohorts, but this is insufficient to establish comparability according to the “Newcastle–Ottawa Quality Assessment Form for Cohort Studies” between outcomes. Despite the overall poor outcome, the studies scored high on patient selection, showing that the preferred study population was represented well. All studies reported on the ascertainment of exposure and demonstration of the outcome, also eight out of nine scored on the representativeness of the cohort. In addition, little to no bias was found while assessing study outcomes, as demonstrated in Table 5.

## Discussion

The present study assessed the outcomes of over 520 open tibial fractures treated primarily at a limb reconstruction center compared to transferred patients. The majority of fractures were classified as GA grade III-A or higher. The results from our meta-analysis suggest that direct admission to a specialized center decreases the risk of overall and deep infections, including secondary amputations. Patients treated after HTP underwent fewer surgical procedures compared to the GTP group, performing more one-stage procedures with simultaneous soft tissue closure in an orthoplastic approach. Patients who were transferred had to wait longer for final soft tissue closure and were admitted to the hospital for an average of eight more days. The main reasons for the delay in treating open tibia fractures include a shortage of beds in tertiary centers and delays in accessing the operating theater. However, delay in communication and lack of experience with open tibial fractures due to the relatively low incidence and consequent inadequate assessment and debridement have also been reported [22, 24, 28].

The present review affirms that open tibial fractures are best treated directly in limb reconstruction centers to achieve timely treatment and prevent infectious complications. Moreover, this current review suggests that the total number of surgical interventions was decreased following direct admission. Two studies suggest that HTPs need less interventions in a timeframe up and until 12 months [12, 25]. The additional interventions in TPs can be explained by two combined factors. First, GTP generally undergoes staged procedures, with more bone and soft tissue debridements and a longer time till definitive fixation. Consequently, leading to delayed definitive soft tissue coverage and increased infections [29, 30]. Second, transferred patients, who underwent initial surgical management in a non-orthoplastic hospital, show high revision rates up to 42% [24, 26]. These numbers show that regular transfers need more interventions. These additional interventions include fracture re-fixation, further debridement due to infection, and secondary treatment in non- or malunions [12].

Certain logistical and patient-related factors remain important as they directly affect adherence to the standards and subsequent quality of healthcare. Despite the implementation of national UK guidelines, timely treatment at limb reconstruction centers is persistently seen as challenging, as time of presentation (day versus nighttime) was recently shown to be associated with a longer time to surgery [15]. Additionally, the availability of orthoplastic services played a role in soft tissue coverage delays, and older patients were less likely to be treated in non-MTCs [15, 31]. Furthermore, the time it takes for a patient to be transferred adds up to the total LOHS and could delay definitive surgical management. The overall adherence to UK clinical guidelines has been improving. However, a recent study reveals that six out of 13 standards are not being met, and over 60% of patients are not receiving treatment in orthoplastic centers. [31]. The Trauma Audit and Research Network (TARN) data showed that even in dedicated limb reconstruction centers, the soft tissue coverage standard within 72 h is not always attained. Early referral has in the past demonstrated a drop in hospital expenses and an improvement in overall clinical outcomes [32].

To support the need and necessities to transfer, a Major Trauma Network (MTN) was installed in 2010 in the UK “to ensure that patients with significant injuries are taken promptly to a Major Trauma Center (MTC) where there are specialist services available to treat these patients optimally” [33]. A British study performed in 2018 shows that about one-fifth of all patients with fractures are transferred to another center for definitive treatment [29]. The National Institute for Health and Care Excellence refers in their guideline on complex open fractures of long bones as always being entitled to direct MTC referral [8]. Studies show that the network reduces inter-hospital transfer time by seven days, which is confirmed by our present review. Prior to introducing the MTN, the average number of surgical interventions dropped significantly from 4.2 to 2.3 per patient [34]. A large-scale study has shown that well-established clinical networks generate overall better outcomes and access to healthcare [35].

It is, however, not clear whether the introduction of the MTN leads to “over triage” of open tibial fractures, where patients with GA type I tibial fractures without concomitant injuries have to travel long distances to Level I centers. Moreover, overcrowding in specialized centers is unwarranted and could lead to unnecessary costs and the occupation of valuable theater time [33, 36]. The literature already demonstrated that after the introduction of the major trauma centers in the United Kingdom, the overall plastic surgical operative workload increased sevenfold. This is mainly due to the fact that joint orthoplastic service is now seen as the standard and not as a delayed phase of open fracture management, leading to an increase in extremity referrals [37]. To address concerns, there is a need for a more comprehensive understanding of the specific reasons for patient transfers and the establishment of consensus regarding the fractures that require specialized treatment. The allocation of sufficient funding to support specialized limb reconstructive centers is imperative to organize and coordinate services accordingly.

### ‘Hot transfer’

The alternative to direct admission is transferring stable patients as (“hot transfers”) after first admission in a non-MTC without initial surgical management. As there is no need to perform a first debridement in non-severely contaminated wounds within six hours, this could reduce overcrowding and potentially unnecessary, costly level I treatment [38]. Using photographs during primary clinical treatment can aid decision-making for tertiary referral. Primary debridement can be performed, after which the most appropriate treatment pathway is planned by the limb reconstruction unit receiving the referral. There is, however, a delicate balance between waiting to transfer and initiating treatment. A study conducted in 2019 compared two cohorts with open tibial fractures who received final soft tissue coverage within and after seven days. Of the 672 included patients, two-thirds received final treatment after seven days. Compared to the early group, the risk of developing complications (deep infection, osteomyelitis, and amputation) increased significantly. Thus, it is important to evaluate timely debridement and time till soft tissue coverage as two separate concepts, as the latter calls for an orthoplastic team with allocated OR time and personnel, taking up substantial OR time when free tissue transfer is required. These numbers show that regular transfers need more interventions. These additional interventions include fracture re-fixation, further debridement due to infection, and secondary treatment of nonunions or malunions [12].

### Limitations

The main strength of this review was its narrow focus and comprehensive search. However, several limitations exist. One limitation is that the included articles did not provide sufficient information on the transfer process. Reasons for transfer are not listed, and information such as AO fracture classification distribution, comorbidity, and other injuries is often not mentioned. Moreover, the studies were of overall poor quality according to the NOS—quality assessment scale, with the majority not correcting for confounders such as age or injury severity score (ISS). The level of comparability can create bias on a clinical selection and indication level, as there was no tool to determine the ISS of the cases transferred and directly admitted. This was especially significant in the HTP group, as injury severity plays a vital role in the ability to treat and transfer. The HTP group was also smaller, with only two articles, partially due to inseparable data. A systematic review performed in 2019 on the outcomes of tibia fractures has already brought this problem to light and stated that “there was considerable heterogeneity and lack of detail in the description of the simplest outcomes, such as union, infection, or reoperation” [39]. However, we were able to review the treatment and patient outcomes for transferred versus directly admitted patients. We specified the available results according to the delay in transfer, before or after primary surgical treatment. The results show the differences in clinical outcomes between the groups and shed light on the potential risks of (late-) transfer. Further prospective population-based studies that address the reasons for transferring and correlate patient outcomes to patient characteristics, treatment details, and hospital levels are needed.

## Conclusion

Severe open tibia fractures are complex injuries in need of specialized care. This review highlights the importance of timely treatment in specialized orthoplastic centers as a means to the reduce length of hospital stay and incidence of fracture-related infections. The findings of this review further reinforce the consensus on direct admissions instead of referral in severe open tibia fractures and provide supporting evidence for adherence to BOAST guidelines.

## Data Availability

Data that support the findings of this systematic review are available within the article. Individual study data and search strings are available from the corresponding author on request.

## Abbreviations

GA: Gustilo-Anderson classification
BOA: British Orthopaedic Association
BAPRAS: British Association of Plastic, Reconstructive, and Aesthetic Surgeons
PRISMA: Preferred Reporting Items for Systematic Reviews and Meta-Analysis
DAP: Directly Admitted Patients
HTP: Hot Transfer Patients
GTP: General Transfer Patients
NOS: Newcastle-Ottawa Scale
MTN: Major Trauma Network
MTC: Major Trauma Center
TARN: Trauma Audit and Research Network
